# A New Hybrid Gyroscope with Electrostatic Negative Stiffness Tuning

**DOI:** 10.3390/s130607121

**Published:** 2013-05-30

**Authors:** Bo Yang, Yumei Guan, Shourong Wang, Qi Zou, Xian Chu, Haiyan Xue

**Affiliations:** 1 School of Instrument Science & Engineering, Southeast University, Nanjing 210096, China; E-Mails: srwang@seu.edu.cn (S.W.); xhy_30@163.com (H.X.); 2 Key Laboratory of Micro-Inertial Instrument and Advanced Navigation Technology, Ministry of Education, Nanjing 210096, China; 3 Beijing Aerospace Control Device Institute, Beijing 100854, China; E-Mails: gs_hill@sohu.com (Y.G.); blue77aki@gmail.com (Q.Z.); michaelzhao1978@yahoo.com.cn (X.C.)

**Keywords:** Hybrid Gyroscope (HG), negative stiffness, tuning, rebalancing control loop

## Abstract

A variety of gyroscopes have been extensively studied due to their capability of precision detection of rotation rates and extensive applications in navigation, guidance and motion control. In this work, a new Hybrid Gyroscope (HG) which combines the traditional Dynamically Tuned Gyroscope (DTG) with silicon micromachined technology is investigated. The HG not only has the potentiality of achieving the same high precision as the traditional DTG, but also features a small size and low cost. The theoretical mechanism of the HG with a capacitance transducer and an electrostatic torquer is derived and the influence of the installation errors from the capacitance plate and the disc rotor module is investigated. A new tuning mechanism based on negative stiffness rather than the traditional dynamic tuning is proposed. The experimental results prove that the negative stiffness tuning is practicable and a tuning voltage of as high as 63 V is demonstrated. Due to the decreased installation error, the non-linearity of the scale factor is reduced significantly from 11.78% to 0.64%, as well as the asymmetry from 93.3% to 1.56% in the open loop condition. The rebalancing close-loop control is simulated and achieved experimentally, which proves that the fundamental principle of the HG is feasible.

## 1. Introduction

As an important inertial sensor, the gyroscopes which are used to detect the rotation rate have been a hot researching topic since its emergence. A variety of gyroscopes, such as Traditional Mechanical Gyroscope (TMG), Electrostatic Suspended Gyroscope (ESG), Ring Laser Gyroscope (RLG), Fiber Optic Gyroscope (FOG), Dynamically Tuned Gyroscope (DTG), as well as Silicon Micro-Gyroscope (SMG), have been developed. With the merits of small volume, light weight, low cost, mass production, and high endurance to shock and vibration, SMGs have received great attention in the last twenty years [1]. However, the speed of SMGs is provided by the resonant movement, which makes the moment of momentum of SMGs change periodically over time and limits the improvement of the performance of SMGs. The detection capacitances of SMGs, whose typical values are in the order of an aF or even lower, are so weak that they are susceptible to the influence of the surrounding parasitic capacitances and coupling noise. In addition, other influences, such as the thin proof mass, once only shaping, residual stress and fabrication tolerance, also restrict the improvement of the precision of SMG. The SMG is still in a state of development with a lower precision around 0.1∼100°/h which cannot meet the demand in high precision of tactical weapons and needs long-term development to achieve the higher precision [2–6].

The classical gyroscopes, such as the DTG, have high precision (the bias stability is usually around 0.001∼1°/h), but these gyroscopes are large in volume, heavy in weight, costly (they may cost tens of thousands of dollars), weak in endurance to shock and vibration, and complicated in process and assembly [7–9]. Thus, a small, light and low-cost gyroscope whose performance can match that of the expensive traditional gyroscopes is urgently needed.

To meet these requirements, the Hybrid Gyroscope (HG) is a hot research topic. The research on the microelectromechanical hybrid gyroscope was first proposed by Jenkins *et al.* in 2003 when only theoretical analysis but no experiment results was shown [10]. The new HG integrates the theory of the traditional DTG with the silicon micromachined technology. The high-frequency vibration-driven approach commonly used in SMG is abandoned while the high-speed rotary drive is adopted, which effectively overcomes the drawbacks of moment of momentum change over time in the SMG. The fabrication of the HG combines the conventional precision machining with the micro-machining technology. As a result, it is feasible to obtain the same level precision as the traditional DTG with the HG, while maintaining low cost and small volume, which makes it competitive in the market.

## 2. Structural Design and Dynamics

**Figure 1.**
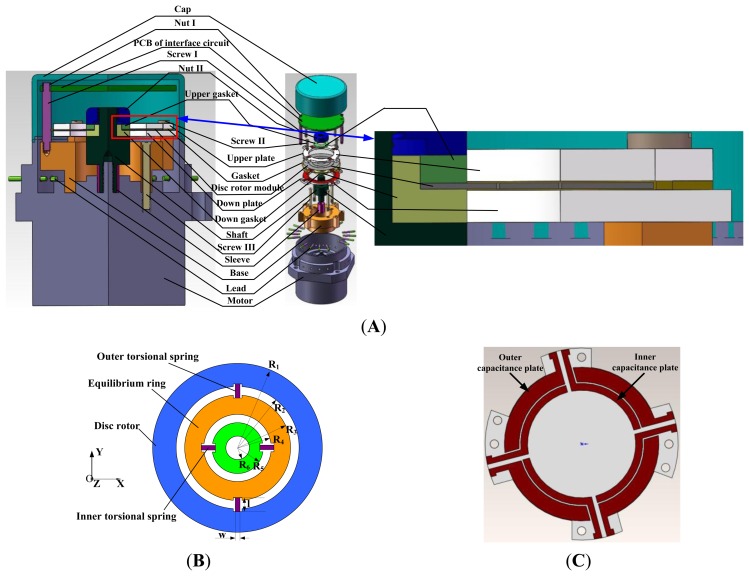
The schematic diagram of the HG. (**A**) Assembly schematic of HG. (**B**) Schematic diagram of the disc rotor module. (**C**) Schematic diagram of the down plate.

The HG structure is designed to verify the basic principle. The HG as shown in Figure 1 consists of a motor, a shaft, a base, an upper/down plate, a silicon disc rotor module, an upper/down gasket, a cap, an interface circuit and so on. The capacitance transducer and the electrostatic torquer are constituted with the upper/down plate and silicon disc rotor module. Different from the traditional DTG using the inductor transducer and the permanent magnetic moving iron torquer, the capacitance transducer commonly used in the SMG is utilized to detect the displacement signal. The electrostatic torquer is exerted to apply to a feedback moment in the HG. By making use of the capacitance transducer and electrostatic torque, we can get rid of several shortcomings including bulky volume, heavy mass and heat effects of the inductor transducer and the permanent magnetic moving iron torquer.

Compared with the traditional DTG, the disc rotor module is adopted simultaneously, which greatly simplifies the rotor structure and the flexible joint in the traditional DTG. Besides, the mass and volume of the rotor with sheet structure are greatly reduced, and the impact resistance and the start-up characteristics are greatly improved. In addition, the silicon disc rotor module and glass upper/down plate which are fabricated in volume through the silicon micro-machining technology do not require assembly.

The disc rotor module of the HG is driven by the electrical motor to obtain a large momentum moment along the z axis. When a rotational rate along X-axis or Y-axis is input, the rotating axis of the disc rotor is deflected due to the Coriolis force, changing the capacitance between the disc rotor and the upper/down capacitor plates. This capacitance change can then be measured to calculate the input rotational rate. To maintain the equilibrium position of the disc rotor, the electrostatic feedback moment is exerted by electrostatic torquer to generate the revising and compensating effects, which is used to indirectly measure the input rotational rate of HG. Neglecting some secondary factors such as damping moments and second order harmonic moment, the motion equations of HG can be expressed as [11]:

(1) $$J\ddot{\gamma }+c\dot{\gamma }+\Delta K\gamma +H\dot{\delta }+\beta \delta =M_{x}-J{\ddot{\phi }}_{x}-H{\dot{\phi }}_{y}$$

(2) $$J\ddot{\delta }+c\dot{\delta }+\Delta K\delta -H\dot{\gamma }-\beta \gamma =M_{y}-J{\ddot{\phi }}_{y}+H{\dot{\phi }}_{x}$$

where H is the momentum moment of HG, J = J_e_ + I_e_/2, J_e_ is the moment of inertia of disc rotor around X-axis or Y-axis , and I_e_ is the moment of inertia of equilibrium ring around X-axis or Y-axis; ΔK is the remaining stiffness; γ and δ are the angular displacements in the rotation axis of the disc rotor along the X-axis and Y-axis respectively; *ϕ̇_y_* and *ϕ̇_x_* are respectively the input rotational rate along OX-axis and OY-axis; M_x_ and M_y_ are respectively the feedback moments of rotor along X-axis and Y-axis, c and β are viscous damping coefficient of the flexible torsion spring and orthogonal damping elasticity coefficient. From Equations (1) and (2), the HG is a two-degree freedom gyroscope and the input rotational rates along X-axis and Y-axis can be detected at the same time.

**Figure 2.**
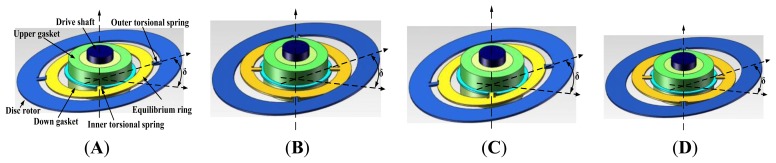
Schematic diagram of torsional motion of disc rotor module. (**A**) θ = 0°. (**B**) θ = 90°. (**C**) θ = 180°. (**D**) θ = 270°.

The structure parameters are shown in Table 1, where h_1_ and h_2_ are the thicknesses of disc rotor module and the inner/outer torsional spring, K_p_ is the torsional rigidity of the inner/outer torsion spring and K_f_ is the flexural rigidity of the inner/outer torsion spring. The disc rotor module of the HG is driven by the electrical motor to rotate rapidly. When a rotational rate along X-axis or Y-axis is input, the disc rotor is deflected around the drive axis and the equilibrium ring is driven to turn over by the disc rotor simultaneously, as shown in Figure 2. At the starting position, when θ = 0°, the inner torsional spring is vertical to the paper, while the outer torsional spring is horizontal to the paper. The flexural rigidity of the outer torsion spring is much larger than the torsional rigidity of the inner torsion spring (shown in Table 1), therefore the disc rotor and the equilibrium ring are driven together to turn a litter angle δ along the axis of inner torsional spring, as shown in Figure 2(A). When the drive axis is turned 90°, the outer torsional spring is vertical to the paper, while the inner torsional spring is horizontal to the paper. The flexural rigidity of the inner torsion spring is much larger than the torsional rigidity of the outer torsion spring (shown in Table 1), hence only the disc rotor is driven to turn a litter angle δ along the axis of the outer torsional spring, as shown in Figure 2(B). It can be analyzed similarly when θ = 180° and θ = 270°.

**Figure 3.**
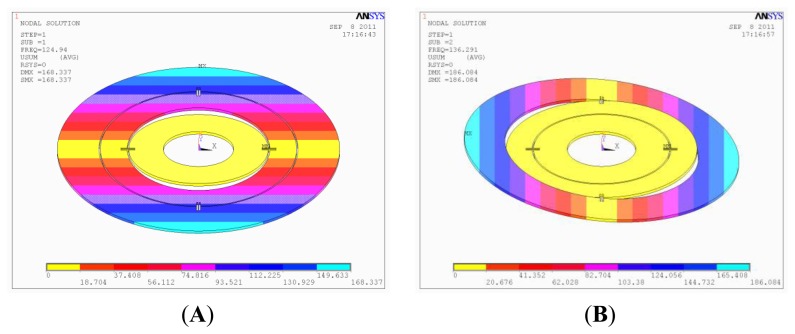
The mode simulation of disc rotor module. (**A**) Rotational mode of inner torsional spring. (**B**) Rotational mode of outer torsional spring.

The disc rotor module is the core part of the HG and directly determines the performance of the gyroscope. The disc rotor is simulated and optimized by ANSYS. Table 2 shows the first six modes of the optimized disc rotor. It can be concluded from Table 2 that the first two useful modes have a preferable isolation with the latter four interfering modes. Meanwhile, the simulation shows that the rotational velocity (or frequency) of the electrical motor should be kept away from the two-order useful frequencies mentioned above. Figure 3 shows the mode of the first two modes. It can be seen that the first two useful modes are the torsional modes of the inside torsional spring and outside torsional spring, which is accordant with the theory. When the angular velocity is input, the disc rotor module convert between the two modes with the rotation of the motor.

**Figure 4.**
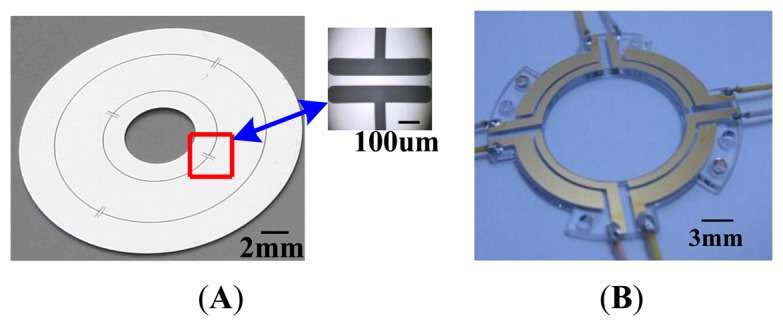
The prototypes of disc rotor module and capacitor plates. (**A**) Disc rotor module. (**B**) Down capacitor plate.

In order to simplify the process, a 4-inch P-type single crystalline silicon wafer with 210 um thickness is adopted. The photoresist (PR) is coated on the wafer to form the mask of disc rotor module. The standard Deep Reactive Ion Etching (DRIE) process with 20:1 aspect ratio is used for fabrication of disc rotor module. Six disc rotor modules can be processed in a 4-inch silicon wafer. Similarly, the capacitor plate can be fabricated first by laser cutting. Then a layer of metal is sputtered on the glass to form capacitor electrodes. The thickness of the capacitor plate is 1 mm. Figure 4 shows the prototype of the disc rotor module and capacitor plates fabricated by a standard microfabrication process.

## 3. The Capacitance Transducer and Electrostatic Torquer

### 3.1. The Capacitance Transducer

**Figure 5.**
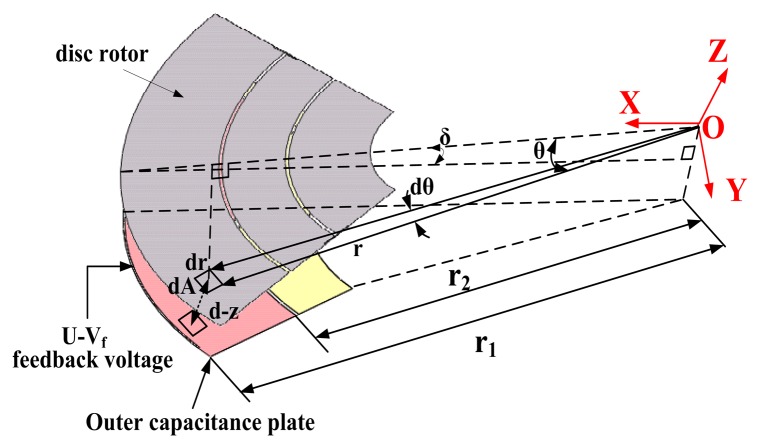
Schematic diagram of disc rotor deflection.

The strength of the detection signal, which is an important parameter in the system simulation and interface circuit design, is directly determined by the capacitance transducer. Suppose the disc rotor turns a small angle δ around the Y axis when the Coriolis force is input, as shown in Figure 5.

Select the infinitesimal dA in the disc rotor, where θ is an angle from infinitesimal to X-axis, and r is the distance from the infinitesimal to center dot O. Therefore the capacitance between the infinitesimal dA and the down plate can be expressed as:

(3) $$\begin{array}{ccc} C_{1}=\iint \frac{ɛr}{d-z}d\theta dr & \text{z}\approx \text{r}\cos\theta \delta ,-\frac{\pi }{4}\leq \theta \leq \frac{\pi }{4}, & {\text{r}}_{2}\leq \text{r}\leq {\text{r}}_{1} \end{array}$$

where d is the static direction from disc rotor to outer capacitance plate, and z is the displacement of the infinitesimal dA along the Z-axis when the disc rotor turn the angle δ around the Y-axis. Similarly, the differential capacitance in the contrary direction is:

(4) $$C_{2}=\iint \frac{ɛr}{d+z}d\theta dr$$

Thus:

(5) $$\Delta C=C_{1}-C_{2}\approx A\delta +B\delta^{3}$$

where

(6) $$\begin{array}{cc} A=\frac{2\sqrt{2ɛ}}{3d^{2}}(r_{1}^{3}-r_{2}^{3}), & B=\frac{2\sqrt{2ɛ}}{3d^{4}}(r_{1}^{5}-r_{2}^{5}) \end{array}$$

where the first item is utilized to convert the angle signal into the capacitance signal, and the second item is the main source of nonlinearity. Therefore, the strength and quality of the detection signal can be determined according to Equation (5).

### 3.2. The Electrostatic Torquer

Like the traditional DTG, the new HG will be an ideal two-degree-of-freedom gyroscope under ideal tuning conditions. When the angular velocity is presented, the disc rotor will precess due to the Coriolis effect. The entire system is consequently unstable and the closed-loop control must be employed. The electrostatic force derived from the capacitive torque of the new HG is used for the closed-loop control. The electrostatic torquer is small in size and light in weight with extremely small heat dissipation effects. Therefore the micro-miniaturization of HG is feasible in practice. The outer capacitance plate is both the capacitance transducer and the electrostatic torquer. When the feedback voltage U-V_f_ is exerted on the outer capacitance plate as shown in Figure 5, the electrostatic torquer along y-axis is:

(7) $$\begin{array}{c} M_{y1}=2\iint dF_{e}•r\cos\theta =\iint \frac{1}{2}\frac{\partial (dC)}{\partial z}{\left(U-V_{f}\right)}^{2}•2r\cos\theta =ɛ{\left(U-V_{f}\right)}^{2}\iint \frac{r^{2}\cos\theta }{{\left(d-r\cos\theta \delta \right)}^{2}}d\theta dr\\ \approx ɛ{\left(U-V_{f}\right)}^{2}(\frac{\sqrt{2}(r_{1}^{3}-r_{2}^{3})}{3d^{2}}+\frac{\left(r_{1}^{4}-r_{2}^{4})(\pi +2\right)}{8d^{3}}\delta +\frac{\sqrt{2}(r_{1}^{5}-r_{2}^{5})}{2d^{4}}\delta^{2}) \end{array}$$

Similarly, the feedback voltage U+V_f_ is exerted on the differential outer capacitance plate, then the consequent electrostatic torquer is

(8) $$M_{y2}\approx -ɛ{\left(U-V_{f}\right)}^{2}(\frac{\sqrt{2}(r_{1}^{3}-r_{2}^{3})}{3d^{2}}-\frac{\left(r_{1}^{4}-r_{2}^{4})(\pi +2\right)}{8d^{3}}\delta +\frac{\sqrt{2}(r_{1}^{5}-r_{2}^{5})}{2d^{4}}\delta^{2})$$

The total electrostatic torquer exerted on the disc rotor is

(9) $$M_{y}=M_{y1}+M_{y2}\approx K_{f}V_{f}+K_{Uf}\;\delta +K_{\text{NUf}}V_{f}\;\delta^{2}$$

where *K_f_* is the torquer coefficient and $K_{f}=-4\sqrt{2ɛ}(r_{1}^{3}-r_{2}^{3})U/(3d^{2})$, *K_Uf_* is the negative stiffness coefficient and $K_{Uf}=(\pi /4+0.5)ɛ(r_{1}^{4}-r_{2}^{4})(U^{2}+V_{f}^{2})/d^{3}$, *K*_NUf_*V_f_* is the nonlinearity coefficient and $K_{\text{NUf}}=-2\sqrt{2}ɛ(r_{1}^{5}-r_{2}^{5})U/d^{4}$. Besides, the first term in Equation (9) is the feedback electrostatic torquer which is mainly used for compensating the gyroscope moment caused by the input rotation rate; the second term in Equation (9) is the negative stiffness moment which changes the effective stiffness of the system. Therefore, according to the Equation (9), the relationship between the feedback voltage V_f_ and torque, as well as the relationship between the bias voltage U and the negative stiffness effect can be determined, which is important to the design of closed-loop control system and negative stiffness dynamic tuning.

## 4. The Installation Error Analysis

### 4.1. The Installation Error of the Capacitance Plate

**Figure 6.**
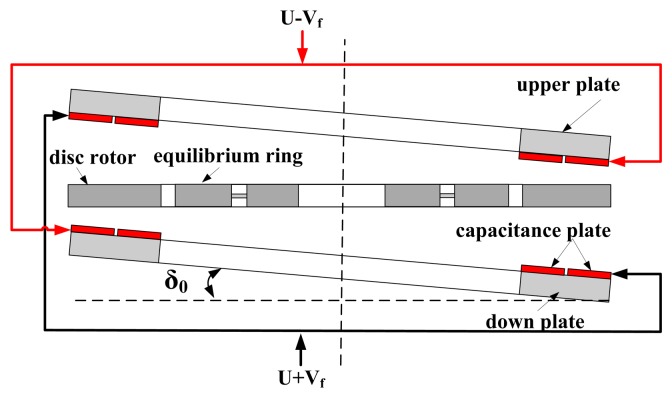
Schematic diagram of the installation error of the capacitance plate.

Since the the down/upper capacitance plate is extremely thin, it is susceptible to installation error which will affect the accuracy of the capacitance transducer and the capacitance torquer. Suppose the capacitance plate has an initial installed deflection angle δ_0_ along the positive deflection direction of the disc rotor as shown in Figure 6. When the disc rotor turns an angle δ around the positive direction since the external rotational rate is input, the deflection angle changes into δ_0_ + δ, according to Equation (5):

(10) $$\Delta C_{P}=[A\delta_{0}+B\delta_{0}^{3}]+[A+3B\delta_{0}^{2}]\delta +3B\delta_{0}\delta^{2}+B\delta^{3}$$

Similarly, when the disc rotor turns an angle δ around the negative direction, the deflection angle changes into δ_0_-δ:

(11) $$\Delta C_{N}=[A\delta_{0}+B\delta_{0}^{3}]-[A+3B\delta_{0}^{2}]\delta +3B\delta_{0}\delta^{2}-B\delta^{3}$$

It can be concluded that the first item in Equations (10) and (11) will bring about the common mode error. Simultaneously, the symbol of the nonlinear terms in the Equations (10) and (11) is disaccordant with linear terms, which causes the asymmetry in positive and negative scale factors.

According to Equation (9), the electrostatic torquer is:

(12) $$M_{y}\approx K_{Uf}\delta_{0}+(K_{f}+K_{\text{NUf}}\;\delta_{0}^{2})V_{f}+K_{Uf}\delta +2K_{\text{NUf}}V_{f}\;\delta_{0}\delta +K_{\text{NUf}}V_{f}\delta^{2}$$

where the first item in Equation (12) is the static error torquer; the second item in Equation (12) demonstrates that the installed deflection angle error of the capacitance plate will change the feedback coefficient; the fourth item is a coupling item between the feedback torquer and the negative stiffness torquer. Therefore the overlarge deflection angle error δ_0_ will result in the tuning failure and feedback instability.

In summary, the installation error of the capacitance plate will result in the asymmetry of scale factors, the static error torquer and the coupling effect between the feedback torquer and the negative stiffness torquer. The precision installation should be achieved to reduce the angle error δ_0_.

### 4.2. The Installation Error of Disc Rotor Module

**Figure 7.**
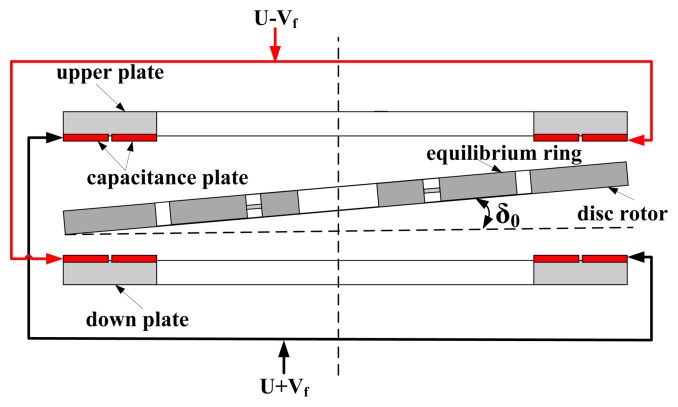
Schematic diagram of the installation error of disc rotor module.

Similarly, the disc rotor module is extremely thin and susceptible to installation errors, which causes the error of the capacitance transducer and the capacitance torquer. Suppose the disc rotor module have an initial installed deflection angle δ_0_, and the external input of rotation rate is zero, as shown in Figure 7. However, the distance between the disc rotor and capacitance plate changes periodically due to the rotation of the rotor. According to Figure 5, the change of the static capacitance is:

(13) $$\begin{array}{ccc} C_{1}=\iint \frac{ɛr}{d-z}d\theta dr & \text{z}\approx \text{r}\cos\theta \delta_{0} & \omega t\leq \theta \leq \omega t+\frac{\pi }{2},{\text{r}}_{2}\leq \text{r}\leq {\text{r}}_{1} \end{array}$$

where ω is the frequency of the rotation rate of the driving motor, ω = 2*πφ̇*/60, and *φ̇* is the rotational velocity of the electrical motor.

The change of the static capacitance is:

(14) $$\Delta C=C_{1}-C_{2}\approx -A\sin(\omega t-\frac{\pi }{4})\delta_{0}-\frac{2\sqrt{2}ɛ(r_{1}^{5}-r_{2}^{5})}{5d^{4}}[\frac{3}{4}\sin(\omega t-\frac{\pi }{4})+\frac{1}{12}\sin(3\omega t+\frac{\pi }{4})]\delta_{0}^{3}$$

It is shown that even if there is no external rotation rate input, the capacitance transducer still has an output error signal. The frequency of the first item of the error output signal is the same as the rotation frequency of the rotor. The error output signal can be reduced by decreasing the deflection angle from the installation error of the disc rotor, δ_0_. Similarly, when the feedback voltage V_f_ = 0, the electrostatic moment is:

(15) $$M_{y}\approx \frac{ɛU^{2}(r_{1}^{4}-r_{2}^{4})\delta_{0}}{d^{3}}(\frac{\pi }{4}-\frac{\sin2\omega t}{2})$$

It can be seen that even if the feedback voltage is zero, the electrostatic torquer still has an error output torquer and the frequency of the second item of the error output torquer is twice the rotation frequency of the rotor.

In summary, the installation error of disc rotor module will result in the output signal error in the rotation frequency of the rotor and the output torquer error in twice the rotation frequency of the rotor. The closed-loop control system should be designed to suppress the above errors.

## 5. The Tuning Mechanism of Negative Stiffness

Tuning is one of the important means to improve performance of the HG. According to Figure 2, the equilibrium ring is driven to rotate along the drive axis and simultaneously turn along the axis of the inner/outer torsion spring, which produces the inertia moment that is applied to the disc rotor. And the inertia moment is opposite with the torsion moment of the inner/outer torsion spring. The remaining stiffness is:

(16) $$\Delta K=K_{p}-K_{n}$$

where K_p_ is the positive torsional rigidity of the inner /outer torsion spring, K_n_ is the negative torque coefficient of inertia moment.

**Figure 8.**
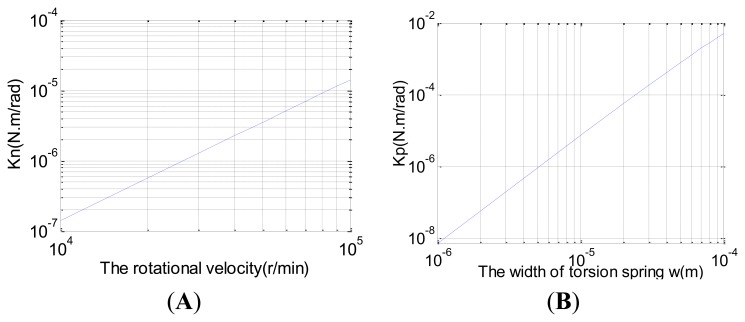
Traditional tuning technique by adjusting the parameters. (**A**) Adjusting the negative torque coefficient by changing rotational velocity. (**B**) Adjusting the positive torsional coefficient by changing the width of torsion spring.

When ΔK = 0, the new HG will beome an ideal two-degree-freedom gyroscope whose mechanical sensitivity is equal to infinity, therefore, optimal performance can be obtained. The traditional DTG is usually tuned by precision machining technology to repeatedly adjust the positive torsional coefficient of the torsional spring K_p_ and the inertia moment of equilibrium ring I_e_ or change the rotation rate of the electrical motor *φ̇*. However, the disc rotor module of the HG is processed by silicon micro-machining technology in only once shaping, which has a relative large fabrication tolerance and is difficult to revise repeatedly thereafter. Thus, the remaining stiffness must exist after the process. Moreover, it's hard to tune by altering the rotational velocity of the motor and the positive torsional coefficient K_p_. The traditional tuning technique by adjusting the parameters is shown in Figure 8. However, issues would occur in practical implementation. Firstly, the rotational velocity of the motor greater than 10,000 r/min is difficult to achieve. Then the thin thickness and width of torsion spring will result in spurious modes and a substantial decline in bending stiffness, which will disable the disc rotor. Typically, the negative torque coefficient of the HG K_n_ is much smaller than the torsional coefficient of the inner and outer torsion spring K_p_ due to the thin thickness (K_n_ < 10^−3^ K_p_). Therefore, the tuning can not be achieved by the tradition implementation method and need be searched for new approaches.

**Figure 9.**
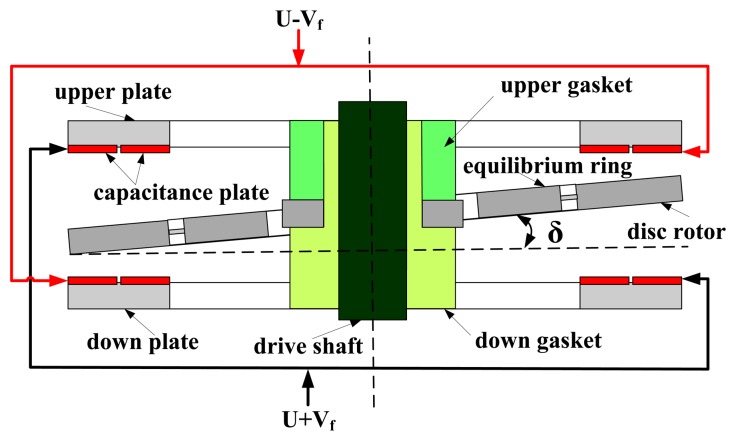
Schematic diagram of the tuning by negative stiffness.

This paper proposes a new tuning method by using the negative stiffness effect of the feedback moment, as shown in Figure 9. It is through exerting a comparatively large preload bias voltage U on the feedback moment plate to generate the negative stiffness moment for tuning.

Substituting Equation (9) into the Equations (1) and (2) and ignoring the error term, we get:

(17) $$J\ddot{\gamma }+c\dot{\gamma }+(\Delta K-K_{Uf})\gamma +H\dot{\delta }+\beta \delta =K_{f}V_{fA}-J{\ddot{\phi }}_{x}-H{\dot{\phi }}_{y}$$

(18) $$J\ddot{\delta }+c\dot{\delta }+(\Delta K-K_{Uf})\delta -H\dot{\gamma }-\beta \gamma =K_{f}V_{fB}-J{\ddot{\phi }}_{y}+H{\dot{\phi }}_{x}$$

where K_f_V_fA_ and K_f_V_fB_ are the feedback moments imposed on the disc rotor along X-axis and Y-axis separately.

Suppose Δ*K*−*K_Uf_* = 0 and U^2^ ≫ *V_f_*^2^, then(the derivation can be seen in the Appendix):

(19) $$\text{U}\approx \pm \sqrt{\frac{d^{3}(\frac{2\alpha Gw^{3}h_{2}}{l}-\frac{1}{12}\pi (R_{3}^{2}-R_{4}^{2})h_{1}^{3}\rho {\dot{\varphi }}^{2})}{ɛ(\pi /4+0.5)(r_{1}^{4}-r_{2}^{4})}}$$

**Figure 10.**
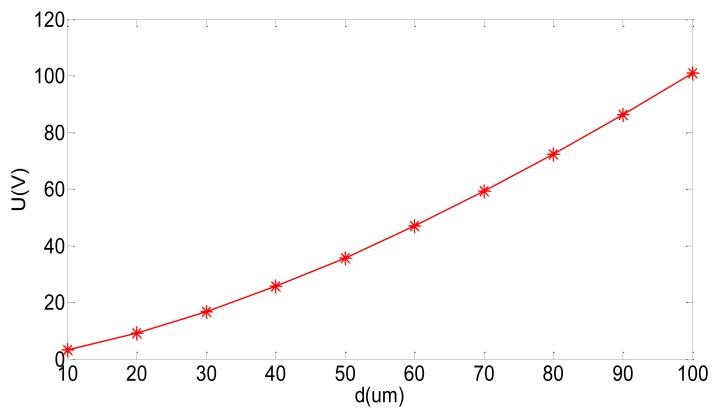
The curve of installation distance d with the tuning voltage U.

The preload bias voltage U used to tune can be calculated by the structural parameters in Equation (19). Since installation errors may present, the changes of installation distance *d* will directly affect the tuning voltage U as shown in Figure 10. To sum up, by adjusting the bias voltage U, the new tuning means can be implemented through the use of the negative stiffness effect in the capacitor torque.

## 6. Design and Simulation of Rebalancing Control Loop

**Figure 11.**
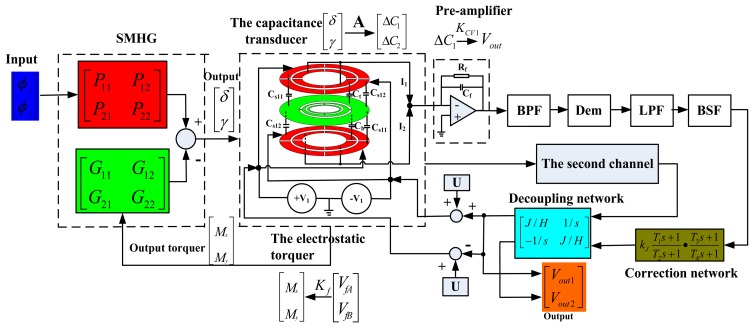
Block diagram of the rebalancing control loop.

The closed-loop control of HG must be achieved under the ideal tuning conditions. The performance of HG can be affected directly by the design of a closed-loop system. The block diagram of the rebalancing control loop system is shown in Figure 11. The symbols P_11_, P_12_, P_21_, P_22_, G_11_, G_12_, G_21_, G_22_ are defined in the Appendix. When the rotational rate *ϕ̇_y_* and *ϕ̇_x_* are input along the OX-axis and OY-axis respectively, the deflection angles of disc rotor γ and δ are output according to the Coriolis effect. Then the deflection angle signal is detected by capacitance transducer. A group of high frequency carrier waves ±V1 with the frequency ω_1_ are applied to a group of outer capacitance plates. At the same time, another group of high frequency carrier waves ±V_2_ with the frequency ω_2_ are applied to another group of capacitance plates. The two groups of sensed differential capacitances are modulated by the two carrier waves in different frequencies (only one interface model is shown in Figure 11, the other interface model would be distributed symmetrically in the same way), and the modulated signals are amplified by the operational amplifier. Then the useful signals are extracted by the Band Pass Filter (BPF), Demodulator (Dem) and Low Pass Filter (LPF). A Band Stop Filter (BSF) is mainly used to suppress the interference signal which is derived from the installation error of the disc rotor module and has the same frequency as the rotation rate of the driving motor ω. Finally, the useful signal which is fed back to the outer capacitance plate is utilized to achieve the rebalancing control by the Correction network and Decoupling network.

Suppose ΔC1 = 2C_s11_ − 2C_s12_, then the induced voltage of the disc rotor is:

(20) $$V_{\text{d}}=\frac{\Delta C_{1}V_{1}}{2C_{s11}+2C_{s12}+C_{t}+C_{b}}$$

so the output voltage of the Pre-amplifier is:

(21) $$V_{\text{out}}=K_{CV1}\Delta C_{1}$$

where *K_CV_*_1_ = (*C_t_*+*C_b_*)*V*_1_/(2*C_s_*_11_ + 2*C_s_*_12_ +*C_t_* + *C_b_*)*C_f_*. Apparently the signal sensitivity can be improved by increasing the capacitances C_t_ and C_b_.

**Figure 12.**
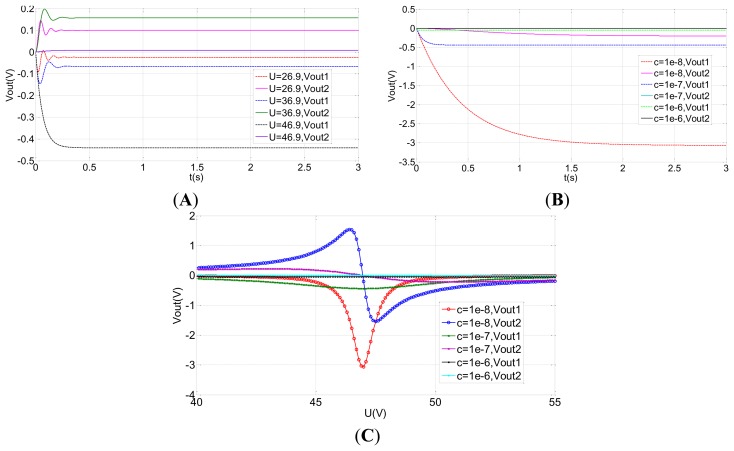
The open-loop system simulation. (**A**) The open-loop output at different tuning voltages. (**B**) The open-loop output with different damping coefficients (U = 46.9 V). (**C**) The curve of output voltage with the tuning voltage in different damping coefficients in the equilibrium state.

In order to optimize the design of the rebalance control loop, the open loop and the closed-loop system is simulated by Matlab. The simulation parameters are shown in Table 3. The correction network and decoupling network are removed in the open loop simulation. The results of the open loop system simulation are shown in Figure 12. Vout1 is the output from the input, *ϕ̇_x_* = 1°/s. Vout2 is the coupling output. It can be concluded from Figure 12(A) that a large coupling signal Vout2 is output and the coupling output Vout2 is greater than the signal output Vout1 when the bias voltage U is far from the tuning voltage point. However, when the voltage U equals to the tuning voltage point 46.9, the coupling output Vout2 is far less than the signal output Vout1. From Figure 12(B), at the tuning voltage point, the smaller the damping is, the greater the output voltage is. Figure 12(C) shows a clear relationship among the tuning voltage, output voltage and the damping coefficient.

**Figure 13.**
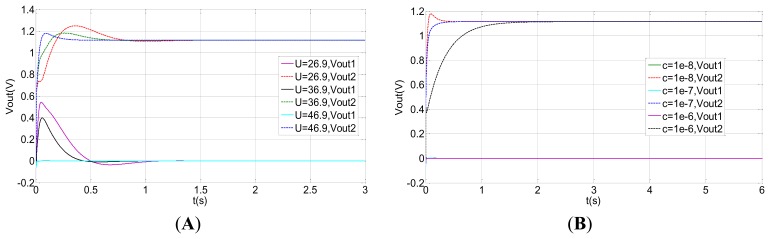
The closed-loop system simulation. (**A**) The closed-loop output at different tuning voltages. (**B**) The closed -loop output with different damping coefficients (U = 46.9 V).

At the same time, the simulation results of the closed-loop system are shown in Figure 13. Nevertheless, the output in the closed-loop system is reversed with those in the open loop system as a result of the Coriolis effect. Vout2 is the output from the input, *ϕ̇_x_* = 1°/s, and the Vout1 is the coupling output. The closed-loop control can be achieved with different damping coefficients and different tuning voltages, illuminating good robustness. Compared with the open loop control, the coupled output in the closed-loop system is basically zero with different damping coefficients and different tuning voltages, which indicates the closed-loop system preserves good decoupling characteristics. From Figure 13(A), the equilibrium process at the tuning voltage point is much quicker than those separated from the tuning voltage point. Under the ideal tuning conditions and without considering damping, the closed-loop system can immediately balance down. Therefore, the equilibration time in the tuning point corresponds primarily to the damping effect. However, apart from the damping effect, the closed-loop system also needs to balance the precession movement from the residual stiffness at non-tuning points. Similarly, the output voltage at the minimal damping coefficient shown in Figure 13(B) has the quickest response velocity.

## 7. Experiments

**Figure 14.**
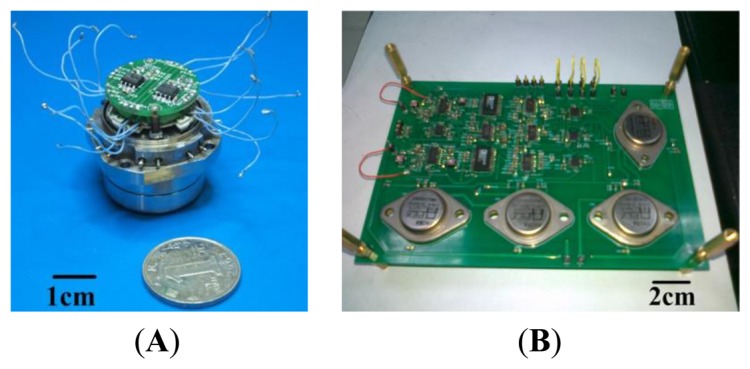
HG Prototype. (**A**) HG structure. (**B**) PCB circuit.

The HG is studied experimentally to verify the principle and the negative stiffness tuning mechanism. In order to simplify the experiment, a traditional hysteresis motor used in the DTG is adopted primarily. A miniature motor based on the micromachining technology will be redesigned to further reduce the volume in the future. The disc rotor module, the down capacitance plate, the motor and other parts are assembled together to form the HG prototype (due to the difficulty in the symmetrical installation of the upper and down plate, only the down plate is installed presently). The upper gasket and down gasket in Figure 1 are adjusted repeatedly to attain an appropriate gap between the down capacitance plate and the disc rotor, as shown in Figure 14(A). According to the principle of rebalancing control loop in the Figure 11, the PCB circuit and interface circuit are designed and debugged, as shown in Figure 14(B).

**Figure 15.**
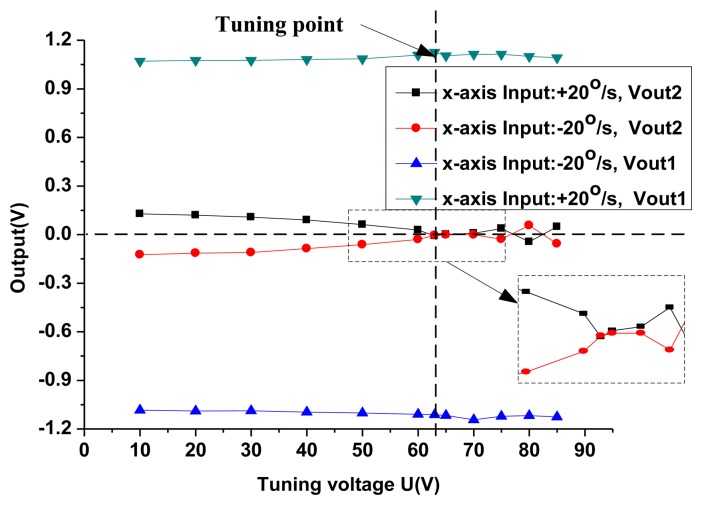
Open-loop tuning experiment.

In the traditional DTG, the tuning point is estimated by the locus of the tip of the gyroscope. However, the tuning point in the new HG is judged by the open loop gain. According to the simulation results in Figure 12(C), the gain difference between the signal output axis and coupling output axis will reach maximum at the tuning point. Consequently, the HG prototype is installed on the rotary table along the x-axis input direction. The input in the x-axis is fixed to ±20°/s in the experiments, and the outputs both in the x-axis (signal axis) and in the y-axis (coupling axis) are measured synchronously at different tuning voltages, shown in Figure 15. When the tuning voltage is increased, the output in the x-axis (signal axis) is essentially unchanged, however, the output in the y-axis (coupling axis) approaches zero gradually. The cross-axis sensitivity is reduced from 11.43% in the 10 V tuning voltage to 0.54% in the 63 V tuning voltage. When the tuning voltage is 63 V, the output in the y-axis (coupling axis) is basically equal to zero. Therefore, the tuning voltage is equal to 63 V (the theoretical value of the tuning voltage in the single plate is 66.3 V). This is very similar to the simulation results in Figure 12(A) when c = 10^−6^. Since the disc-rotor is driven to rotate under the atmosphere with a large damping in the disc-rotor (the vacuum package is not realized).

**Figure 16.**
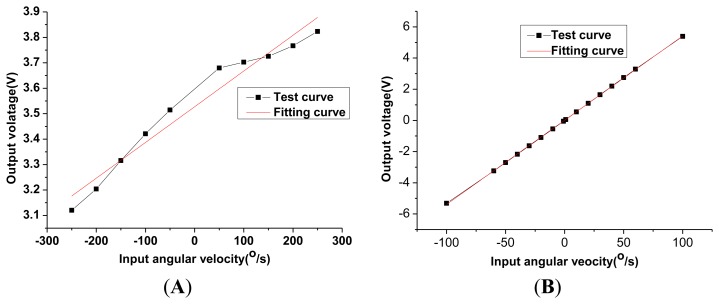
Scale factor comparison test in different installation error. (**A**) The first generation with a large installation error (Open-loop). (**B**) The second generation with a small installation error (Open-loop).

In order to investigate the influence of installation errors, the scale factor for different installation errors are tested experimentally, as shown in Figure 16. Two generations of the HG prototype have been researched. The basic function is implemented in the first generation with a large installation error. When there is no input angular velocity, the gap between the disc-rotor module and the capacitance plate can be observed to change significantly when the disc rotor module rotates one circle. In the second generation, the upper gasket and down gasket is added in the design and other accessories are also redesigned and adjusted accordingly, as shown in Figure 1. At the same time, the optical measurement is utilized to revise the installation errors. By adjusting accurately the dimension of the upper gasket and down gasket, an appropriate gap between the down capacitance plate and the disc rotor can be achieved and most of the installation errors are eliminated. The comparison of the test results is shown in Table 4. It can be seen that the non-linearity and the asymmetry of the scale factor are reduced significantly due to the decreasing in the installation error in the second generation. The test results are consistent with the theoretical analysis results in Section 4.

**Figure 17.**
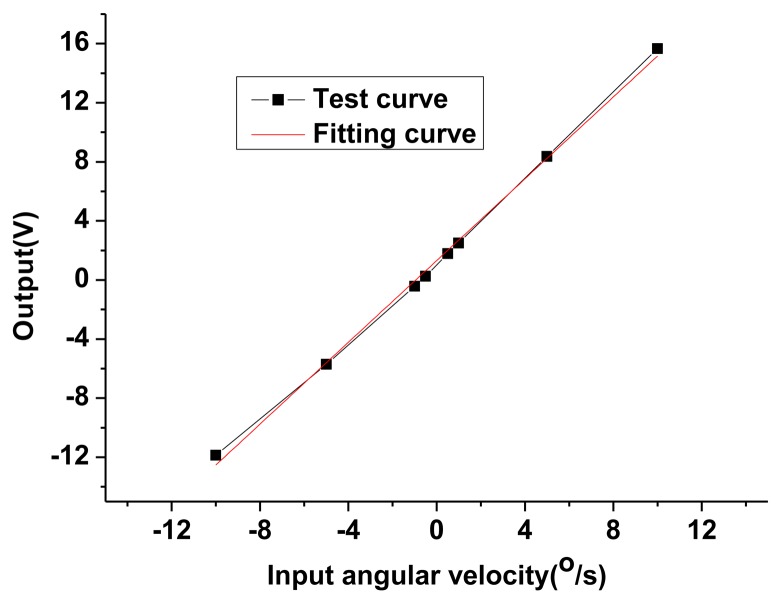
The closed-loop scale factor test of the second generation with a small installation error.

At the tuning voltage point, the closed-loop control is achieved basically by adjusting the loop gain and parameters. The closed-loop scale factor test is carried out, as shown in Figure 17. The experimental results show that the scale factor is 1.3837 V/°/s, the non-linearity and the asymmetry of scale factor are respectively 2.34% and 13.36%. Obviously the non-linearity and the asymmetry of the scale factor in the open loop are better than those in the closed-loop. Although most of the installation errors are corrected with optical methods, some small errors are still difficult to eliminate. The feedback voltage causes different bias voltages in differential capacitor plates. The difference in bias voltage, coupling with the small remaining installation error, results in the torque error which will worsen the non-linearity and the asymmetry of scale factor. However, the influence can be eliminated by further reducing installation errors.

## 8. Conclusions

In this work, a new dynamically tuned HG is investigated. The structure of the new HG which adopts the capacitance transducer and electrostatic torquer is designed. The crucial silicon disc-rotor module is simulated and fabricated on SOI wafers using a standard microfabrication process. The theoretical mechanism of the capacitance transducer and the electrostatic torquer is deduced and analyzed under ideal conditions. Simultaneously the influence of the installation errors of the capacitance plate and disc rotor module upon the performance of capacitance transducer and electrostatic torquer are investigated. A new tuning mechanism based on negative stiffness rather than the traditional dynamic tuning is proposed. The rebalancing closed-loop control scheme is designed, and the open loop tuning mechanism and the closed-loop system are verified by simulation. By assembling and adjusting the HG parts, the principle prototype is realized. The experiment results validate that the negative stiffness tuning is feasible. The comparison in two generation prototypes with different installation error shows that the non-linearity and the asymmetry of the scale factor are reduced significantly by decreasing the installation error. Finally, the rebalancing control loop is achieved, which proves that the fundamental principle of the HG is feasible. Future work includes further reducing installation error, redesigning the miniaturization motor and packaging HG in vacuum.

## Figures and Tables

**Table 1.** The structure parameters.

**Parameter**	**Value**	**Parameter**	**Value**	**Parameter**	**Value**
R_1_ (mm)	10	R_5_ (mm)	4.95	K_p_ (N·m/rad)	6.860 × 10^−4^
R_2_ (mm)	7.05	R_6_ (mm)	2.5	I_e_ (kg·m^2^)	6.4107 × 10^−10^
R_3_ (mm)	6.95	h_1_ (um)	210	J_e_ (kg·m^2^)	2.8928 × 10^−9^
R_4_ (mm)	5.05	l × w × h_2_ (um)	1,000 × 48 × 210	K_f_ (N·m/rad)	0.0096

**Table 2.** The first six modes of the disc rotor module.

**Mode#**	**1**	**2**	**3**	**4**	**5**	**6**
Frequency (Hz)	125	136	989	1,071	1,995	2,333

**Table 3.** The simulation parameters.

**Parameter**	**Value**	**Parameter**	**Value**
r_1_ (mm)	10	*φ̇* (rpm/min)	10000
r_2_ (mm)	8.1	H(rad·kg·m^2^/s)	6.7355 × 10^−6^
d (um)	60	K_N_(N·m/rad)	1.41 × 10^−7^
V1(V)	2.5	U(V) (Tuning point)	46.9
β	5 × 10^−9^	c	1 × 10^−8^
T_1_	0.1	T_2_	0.002
T_3_	0.01	T_4_	0.001
*ϕ̇_x_* (°/s)	1	*ϕ̇_y_* (°/s)	0

**Table 4.** Scale factor comparison test in different installation error.

	**Parameter**	**Scale factor (V/**°**/s)**	**Non-linearity**	**Asymmetry**
The first generation with a large installation error (open-loop)	entire range	0.0014	11.78%	93.3%
	positive direction	0.0007	2.05%	
	negative direction	0.00201	7.99%	

The second generation with a small installation error (open-loop)	entire range	0.05402	0.64%	1.56%
	positive direction	0.05405	0.62%	
	negative direction	0.05321	0.53%	

## Appendix

In order to illuminate the relation between the input and output, the transfer function is derived. According the Equations (1) and (2), the motion equations of Hybrid Gyroscope are

(22) $$J\ddot{\gamma }+c\dot{\gamma }+\Delta K\gamma +H\dot{\delta }+\beta \delta =M_{x}-J{\ddot{\phi }}_{x}-H{\dot{\phi }}_{y}$$

(23) $$J\ddot{\delta }+c\dot{\delta }+\Delta K\delta -H\dot{\gamma }-\beta \gamma =M_{y}-J{\ddot{\phi }}_{y}+H{\dot{\phi }}_{x}$$

So the Laplace transform is

(24) $$\left[\begin{array}{c} \gamma \\ \delta \end{array}\right]=\left[\begin{array}{cc} G_{11} & G_{12}\\ G_{21} & G_{22} \end{array}\right]\;\left[\begin{array}{c} M_{x}\\ M_{y} \end{array}\right]-\left[\begin{array}{cc} P_{11} & P_{12}\\ P_{21} & P_{22} \end{array}\right]\;\left[\begin{array}{c} \phi_{x}\\ \phi_{y} \end{array}\right]$$

where

(25) $$\left[\begin{array}{cc} G_{11} & G_{12}\\ G_{21} & G_{22} \end{array}\right]=\left[\begin{array}{cc} \frac{Js^{2}+cs+\Delta K}{\Delta (s)} & -\frac{Hs+\beta }{\Delta (s)}\\ \frac{Hs+\beta }{\Delta (s)} & \frac{Js^{2}+cs+\Delta K}{\Delta (s)} \end{array}\right]$$

(26) $$\left[\begin{array}{cc} P_{11} & P_{12}\\ P_{21} & P_{22} \end{array}\right]=\left[\begin{array}{cc} \frac{J^{2}s^{4}+Jcs^{3}+(J\Delta K+H^{2})s^{2}+H\beta s}{\Delta (s)} & \frac{(Hc-J\beta )s^{2}+H\Delta Ks}{\Delta (s)}\\ -\frac{(Hc-J\beta )s^{2}+H\Delta Ks}{\Delta (s)} & \frac{J^{2}s^{4}+Jcs^{3}+(J\Delta K+H^{2})s^{2}+H\beta s}{\Delta (s)} \end{array}\right]$$

and Δ(*s*) = *J*^2^*s*^4^ + 2*Jcs*^3^ + (*c*^2^ + 2*J*Δ*K* + *H*^2^)*s*^2^ + (2*c*Δ*K* + 2*Hβ*)*s* + Δ*K*^2^ + *β*^2^.

In addition, the tuning voltage of the negative stiffness is deduced. According to the Equation (9), suppose feedback torques along the x-axis and y-axis is respectively.

(27) $$M_{x}\approx K_{f}V_{f\text{A}}+K_{Uf}\gamma$$

(28) $$M_{y}\approx K_{f}V_{f\text{B}}+K_{Uf}\delta$$

Substituting the above equations into the Equations (1) and (2) and ignoring the error term, the new equation are

(29) $$J\ddot{\gamma }+c\dot{\gamma }+(\Delta K-K_{Uf})\gamma +H\dot{\delta }+\beta \delta =K_{f}V_{fA}-J{\ddot{\phi }}_{x}-H{\dot{\phi }}_{y}$$

(30) $$J\ddot{\delta }+c\dot{\delta }+(\Delta K-K_{Uf})\delta -H\dot{\gamma }-\beta \gamma =K_{f}V_{f\text{B}}-J{\ddot{\phi }}_{y}+H{\dot{\phi }}_{x}$$

where the remaining s tiffness is

(31) $$\Delta K=K_{p}-K_{n}$$

The negative torque coefficient of inertia moment K_n_ is

(32) $$K_{n}=(I_{e}-I_{z}/2){\dot{\varphi }}^{2}\approx [\frac{1}{12}\pi (R_{3}^{2}-R_{4}^{2})h_{1}^{3}\rho ]{\dot{\varphi }}^{2}$$

where I_z_ is the inertia moment of equilibrium ring around Z-axis. R_3_ and R_4_ are the outside diameter and the inner diameter of equilibrium ring. h_1_ is the thickness of the equilibrium ring. ρ is the density of silicon. *φ̇* is the rotational velocity of the electrical motor.

The positive torsional coefficient of the inner and outer torsion spring, K_p_ is

(33) $$K_{p}=2\times \alpha \frac{Gw^{3}h_{2}}{l}$$

where h_2_ is thickness of the torsional spring , G is shear modulus, 5.2 × 10^10^. α is relational with h/a.

(34) $$K_{Uf}=(\pi /4+0.5)ɛ(r_{1}^{4}-r_{2}^{4})(U^{2}+V_{f}^{2})/d^{3}$$

Suppose Δ*K* − *K_Uf_* = 0 and ${\text{U}}^{2}\gg V_{f}^{2}$, then

(35) $$\text{U}\approx \pm \sqrt{\frac{d^{3}(K_{p}-K_{n})}{ɛ(\pi /4+0.5)(r_{1}^{4}-r_{2}^{4})}}=\pm \sqrt{\frac{d^{3}(\frac{2\alpha Gw^{3}h_{2}}{l}-\frac{1}{12}\pi (R_{3}^{2}-R_{4}^{2})h_{1}^{3}\rho {\dot{\varphi }}^{2})}{ɛ(\pi /4+0.5)(r_{1}^{4}-r_{2}^{4})}}$$
